# DNA Methylation‐Regulated ZDHHC24 Exacerbates the Risk of Intracranial Aneurysms

**DOI:** 10.1002/brb3.71213

**Published:** 2026-04-06

**Authors:** Hao Yuan, Can Li, Xin Feng, Jiancheng Lin, Chi Huang, Mengshi Huang, Chuanzhi Duan

**Affiliations:** ^1^ Neurosurgery Center, Department of Cerebrovascular Surgery, Engineering Technology Research Center of Education Ministry of China on Diagnosis and Treatment of Cerebrovascular Disease Zhujiang Hospital, Southern Medical University Guangzhou China

**Keywords:** DNA methylation, gene expression, intracranial aneurysms (IAs), Mendelian randomization (MR), palmitoylation

## Abstract

**Purpose:**

Recent studies have reported that palmitoylation plays a pivotal role in the process of angiogenesis, as well as in the motility and migration of endothelial cells (ECs). However, the role of palmitoylation in the pathogenesis of intracranial aneurysms (IAs) has not yet been systematically investigated. Moreover, alterations in DNA methylation and the expression of IA‐related genes have not received sufficient attention.

**Method:**

The present study aims to employ Mendelian randomization (MR) methods to investigate the causal relationships and underlying mechanisms between palmitoylation‐related genes, DNA methylation, and IAs. By employing two‐sample MR analysis, summary‐data‐based Mendelian randomization (SMR) analysis, and mediation analysis, this study drew its conclusions. Furthermore, the robustness of these conclusions was evaluated through sensitivity analyses and transcriptomics approaches.

**Finding:**

Our study revealed a significant positive correlation between the overexpression of the ZDHHC24 gene and increased risk of IA. For each one standard deviation increase in ZDHHC24 expression, the risk of IA increases by 21.85%. Further mediation effect analysis revealed that methylation sites cg01806972, cg10523820, cg18862171, and cg26041493 indirectly influence the occurrence of IA by regulating the expression of the ZDHHC24 gene, with mediation effects accounting for 33.19%, 26.71%, 32.93%, and 47.16% of the total effect, respectively. Sensitivity analysis provides evidence of the robustness of the research conclusion. The final transcriptomic analysis revealed a statistically significant differential expression of ZDHHC24 between experimental and control groups (*p* < 0.001), suggesting its potential involvement in IA pathogenesis.

**Conclusion:**

ZDHHC24 overexpression is causally linked to increased IA risk, and this relationship is mediated by specific DNA methylation loci. These findings underscore the roles of palmitoylation‐related gene expression and DNA methylation in IA pathogenesis, offering novel insights into the molecular mechanisms underlying IA susceptibility.

## Graphical abstract

Multimodal Mendelian randomization integrating multi‐omics datasets pinpointed four DNA‐methylation loci that act in concert with the palmitoylation gene ZDHHC24 to modulate intracranial aneurysm risk, shedding new light on the molecular etiology of IA susceptibility and highlighting potential therapeutic targets.

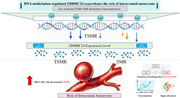

**Abbreviations**
aSAH: aneurysmal subarachnoid hemorrhage
AUC: achieving an area under the curve
DEGs: differentially expressed genes
ECs: endothelial cells
eQTL: expression quantitative trait loci
GBD: Global Burden of Disease
GEO: Gene Expression Omnibus
GO: gene ontology
GoDMC: genome‐wide DNA methylation consortium
GWAS: genome‐wide association study
HEIDI: heterogeneity in dependent instruments
IAs: intracranial aneurysms
ICD: international classification of diseases
IVs: instrumental variables
IVW: inverse‐variance weighted
KEGG: Kyoto Encyclopedia of Genes and Genomes
MAVS: mitochondrial antiviral signaling protein
mQTL: methylation quantitative trait loci
MR: Mendelian randomization
NAFLD: Nonalcoholic Fatty Liver Disease
PTM: posttranslational modification
PWAS: proteome‐wide association studies
ROC: receiver operating characteristic
SMR: summary‐data‐based Mendelian randomization
SNPs: single‐nucleotide polymorphisms
TWAS: transcriptome‐wide association studies

## 1 Introduction

Unruptured IAs affect 3%–5% of adults (Vernooij et al. 2007). Rupture of IAs results in aneurysmal subarachnoid hemorrhage (aSAH), a subtype of stroke that has high mortality and morbidity (Jaja et al. 2018). The increasing use of advanced brain imaging techniques has led to a rise in the detection of incidentally discovered unruptured IAs (Gabriel et al. 2010). While preventive endovascular or neurosurgical treatments offer potential benefits for IAs, these interventions are often accompanied by risks associated with surgical complications. Furthermore, the formation and progression of IAs are influenced by a complex interplay of demographic, genetic, and lifestyle factors, exhibiting dynamic growth characteristics (Villablanca et al. 2013; Brinjikji et al. 2018). This underscores the urgent need to elucidate the mechanistic underpinnings governing aneurysmal risk and progression, and to identify actionable therapeutic targets for IA prevention and treatment.

Recent years have witnessed remarkable advances in genomic and multi‐omics technologies, including GWAS, proteome‐wide association studies (PWAS), and transcriptome‐wide association studies (TWAS). These cutting‐edge approaches have led to the systematic identification of genetic variants and epigenetic mechanisms associated with IA pathogenesis (Wu et al. 2024). Palmitoylation, a reversible posttranslational modification (PTM), involves the covalent attachment of palmitic acid to target proteins and plays a pivotal role in regulating protein function, subcellular localization, stability, and signaling transduction pathways (Linder and Deschenes 2007). Emerging evidence has implicated dysregulated protein palmitoylation in the pathogenesis of various diseases, including tumorigenesis, immune evasion, as well as inflammatory bowel disease and Alzheimer's disease (Chen et al. 2017; Zhang et al. 2020; Zhang et al. 2025). Moreover, accumulating evidence has extensively documented the regulatory roles of palmitoylation in both angiogenesis and the progression of atherosclerosis (Zhou et al. 2025; Ma et al. 2024). However, the precise mechanistic role of palmitoylation in the pathogenesis of IAs remains to be fully elucidated.

Methylation, another pivotal epigenetic modification mechanism, serves as a critical interface between environmental exposures and the genome, while being dynamically regulated by both genetic predisposition and environmental factors (Cilleros‐Portet et al. 2025). Methylation can occur on various biological molecules, including DNA, RNA, proteins, and lipids (Ouyang et al. 2025). DNA methylation can either suppress or activate gene transcription depending on the specific genomic region and degree of methylation, which may exist in mono‐, di‐, or tri‐methylated forms under certain conditions. Consequently, the regulation of intracellular methylation is considerably more complex (Kouzarides 2007; Wu and Zhang 2017). Studies have demonstrated that DNA methylation is involved in the pathogenesis of various diseases, including atherosclerosis, inflammatory bowel disease, and Parkinson's disease (Damiani et al. 2025; Zhang et al. 2025; Liao et al. 2025). Furthermore, research by Chen Lin et al. (2025) has substantiated the involvement of specific DNA methylation loci in the pathogenesis of IAs, although the precise relationship between DNA methylation alterations and the expression of IA‐associated genes remains incompletely understood.

Given that DNA methylation pervasively orchestrates the epigenetic control of diverse cellular processes, it may likewise govern the expression of palmitoylation‐related genes, thereby exerting a latent influence on the risk of developing IAs. To test this hypothesis, we employed a two‐sample MR analysis—a method leveraging genetic variants as instrumental variables (IVs) to determine whether observed exposure‐outcome associations are consistent with causal effects—while rigorously controlling for potential confounding and reverse causation biases (Bowden et al. 2015). We subsequently conducted integrative analysis using the summary‐data‐based SMR method, combining GWAS data of IAs from the Finnish population with blood‐derived expression quantitative trait loci (eQTLs) and DNA methylation quantitative trait loci (mQTLs). This approach enabled systematic prioritization of putative blood‐based palmitoylation‐related genes and their regulatory elements potentially associated with IA risk (Zhu et al. 2016). In addition, we employed mediation analysis to examine the mechanistic relationship between DNA methylation and palmitoylation‐related gene expression in IA, decomposing the total effect into direct and indirect components while quantifying the proportion mediated. Finally, we sought to experimentally validate this hypothesis through systematic analysis of gene expression profiles. These investigations provide novel insights into the epigenetic mechanisms underlying IA pathogenesis, with the potential to illuminate new pathways for early diagnosis and precision therapeutics in IA management.

## 2 Materials and Methods

### 2.1 Summary Data of IA

In the present study, GWAS summary statistics from the R12 release of the Finnish FinnGen database were utilized as outcome data to ensure adequate statistical power for subsequent analytical procedures. The FinnGen database represents a large‐scale collaborative initiative that systematically integrates digital health records from Finnish healthcare registries with genetic data derived from Finnish biobank samples (https://www.finngen.fi/en) (Kurki et al. 2023). The dataset employed in this investigation (ID: finngen_R12_I9_ANEURYSM.gz) comprised 3,310 cases of unruptured IAs and 453,828 control samples. Case ascertainment was performed according to the International Classification of Diseases (ICD) diagnostic criteria, utilizing both the 9th (ICD‐9) and 10th (ICD‐10) revision codes as defined by the Global Burden of Disease (GBD) study. Comprehensive demographic and clinical characteristics of the study population are available through the official database portal.

### 2.2 Summary Data of DNA Methylation

The DNA methylation data were obtained from the GoDMC (Genome‐wide DNA Methylation Consortium) database, which contains comprehensive meta‐analysis results of *cis*‐ and *trans*‐acting mQTLs derived from genome‐wide scans of 420,509 CpG methylation sites. The relevant datasets are publicly accessible at: http://mqtldb.godmc.org.uk/downloads. Regarding palmitoylation‐related genes, the corresponding DNA methylation site information can be retrieved from the EWAS Data Hub at: https://ngdc.cncb.ac.cn/ewas/datahub/exploration.

### 2.3 Summary Data of eQTL

Through a comprehensive literature review, we systematically identified 31 palmitoylation‐associated genes, encompassing members of the PPT family, ZDHHC family, APT family, and ABHD family (Feng et al. 2024; Wang and Yang 2021; Sobocińska et al. 2018). Subsequent interrogation of the eQTLGen consortium database (https://eqtlgen.org) yielded eQTL data for 22 of these candidate genes. Details of candidate genes can be found in Table S1.

The present study exclusively utilized publicly available datasets that had obtained full ethical approval and participant consent during their original collection phases. Consequently, this secondary analysis did not require additional institutional ethics review, as confirmed by prevailing guidelines governing the use of preexisting anonymized genomic data.

### 2.4 IVs Acquisition and Sensitivity Analysis

In the selection of IVs, we applied stringent genomic criteria: (1) only single‐nucleotide polymorphisms (SNPs) demonstrating genome‐wide significant associations with gene expression (*p* < 5 × 10^−8^) were included; (2) all selected IVs exhibited *F*‐statistics > 10 to minimize weak instrument bias; and (3) SNPs in linkage disequilibrium (LD) (*r* ^2^ threshold < 0.001 within 10,000 kb windows) were systematically excluded to ensure genetic independence. Within this analytical framework, we implemented five distinct MR approaches to systematically evaluate the putative causal relationships between palmitoylation‐related gene expression and IA susceptibility. The inverse‐variance weighted (IVW) method served as the primary analytical approach, while supplementary validation was provided through four complementary methodologies: the weighted median estimator, MR‐Egger regression, weighted mode, and simple mode approaches. All MR analyses were conducted using the “TwoSampleMR” package (version 0.5.7) in R version 4.4.1 (Hemani et al. 2018). To manage potential pleiotropy concerns when more than two variants were used as proposed IVs, we employed MR‐Egger regression with Egger intercepts to assess this association (Bowden et al. 2015). For detecting heterogeneity across genetic instruments, Cochran's *Q* test was applied (*p* < 0.05) (Greco et al. 2015). If significant heterogeneity was observed, a random‐effect IVW model was used; otherwise, a fixed‐effect IVW model was favored. Scatter plots were employed to visualize the data, and the leave‐one‐out method was utilized to sequentially exclude individual SNPs to assess their influence on the results.

### 2.5 SMR Analysis

SMR analysis was utilized to estimate the association between DNA methylation and IA (https://cnsgenomics.com/software/smr/). SMR analysis is MR approach in which the genetic variant, DNA methylation and trait are defined as the top‐associated *cis*‐mQTL, exposure, and the outcome, respectively. This analysis follows a two‐step least‐squares approach, incorporating the effect size of the top‐associated *cis*‐mQTL (within a 2‐Mb window) and its corresponding effect in the GWAS. Only probes with a *p* value threshold of 5.0 × 10^−8^ were included to select the top‐associated mQTLs for SMR analysis, as one of the assumptions for MR analysis is that the IV has a strong effect on the exposure. We subsequently performed heterogeneity in dependent instruments (HEIDI) testing to investigate potential heterogeneity within the SMR association statistics.

### 2.6 Mediation Analysis of DNA Methylation–Palmitoylation–IA Relationship

Through integrated MR and SMR analyses, we definitively established: (i) causal relationships between methylation sites and gene expression regulation, (ii) causal effects of gene expression on IA risk, and (iii) aggregate effects of methylation sites on IA susceptibility. Subsequently, we decomposed the total effects into direct and indirect components, with precise quantification of the mediation proportion. Given the fundamental assumption of no unmeasured confounding in mediation analysis, we employed genetic instruments to mitigate potential confounding bias and conducted comprehensive sensitivity analyses to rigorously evaluate the robustness of our findings.

### 2.7 Transcriptomics Analyses

The transcriptomic data utilized in this study were exclusively sourced from the Gene Expression Omnibus (GEO) database, with specific incorporation of datasets GSE26969 and GSE75436. Both datasets were generated using the GPL570 platform, comprising: (i) GSE75436: 15 IA specimens and 15 normal vascular control samples; (ii) GSE26969: 3 IA specimens and 3 matched controls. The Wilcoxon rank‐sum test was ultimately employed to identify differential expression of ZDHHC24 between case and control groups in dataset GSE75436.

## 3 Results

### 3.1 MR Analysis of the Relationship between Palmitoylation and IA

**FIGURE 1:**
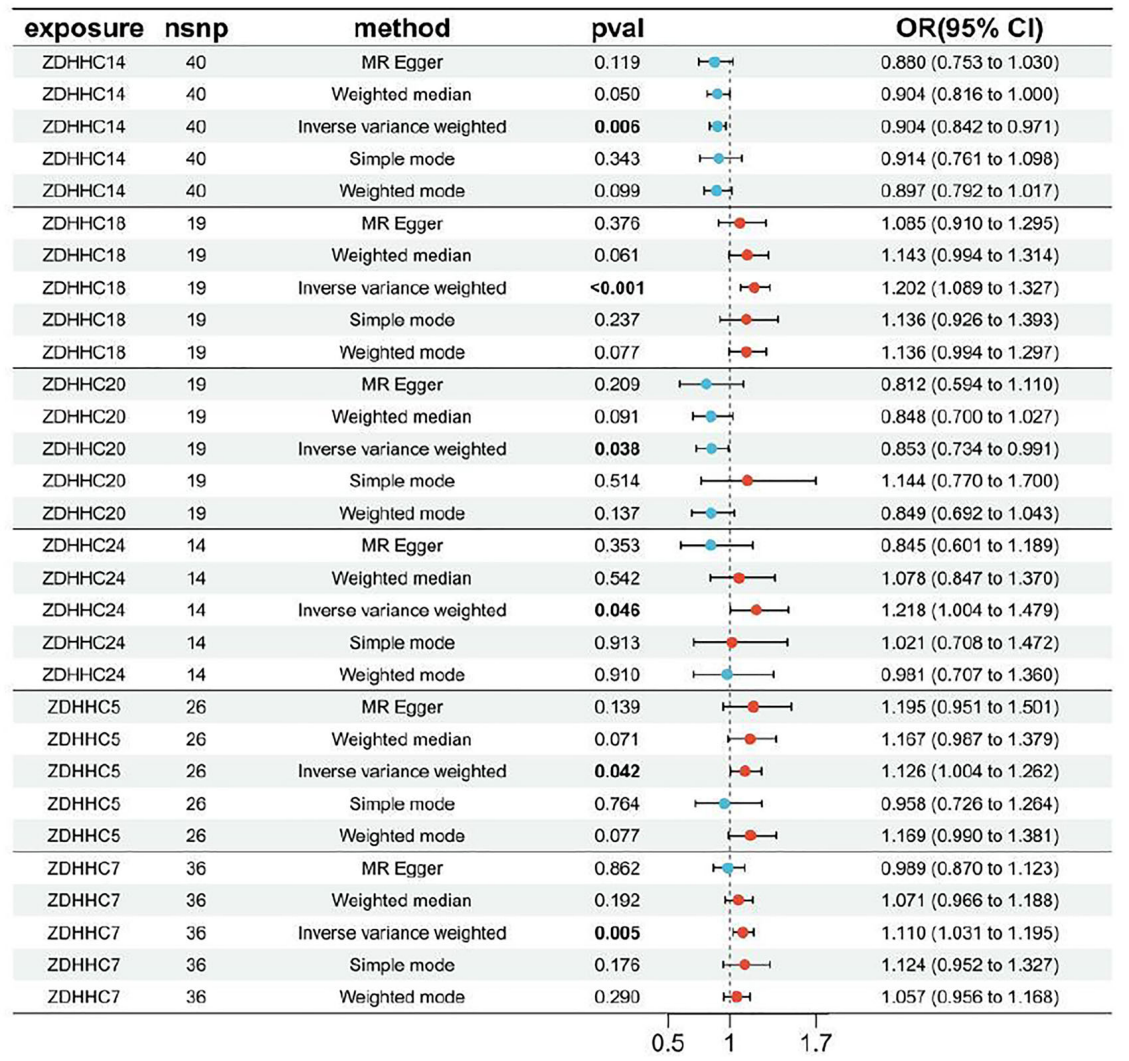
Causal effect of palmitoylation genes on IA risk assessed by IVW method.

Following stringent quality control procedures that eliminated all weak IVs (*F*‐statistic < 10), a final set of 155 SNPs was selected for evaluating the causal effects of palmitoylation‐related genes on IA risk (Table S2). MR Effects for each IV are provided in Table S3. Using IVW as the primary analytical method, we identified six palmitoylation‐related genes demonstrating significant causal associations with IA susceptibility: ZDHHC5, ZDHHC7, ZDHHC14, ZDHHC18, ZDHHC20, and ZDHHC24 (Figure 1). MR analysis identified significant but directionally distinct causal associations between ZDHHC genes and IA risk: ZDHHC14 (OR = 0.904, 95% CI: 0.842–0.971, *p* = 0.006) and ZDHHC20 (OR = 0.853, 95% CI: 0.734–0.991, *p* = 0.038) exhibited protective effects, with increased expression associated with reduced IA risk, while ZDHHC5 (OR = 1.126, 95% CI: 1.004–1.262, *p* = 0.042), ZDHHC7 (OR = 1.110, 95% CI: 1.031–1.195, *p* = 0.005), ZDHHC18 (OR = 1.202, 95% CI: 1.089–1.327, *p* < 0.001), and ZDHHC24 (OR = 1.218, 95% CI: 1.004–1.479, *p* = 0.046) demonstrated risk‐enhancing effects, where elevated expression correlated with increased IA susceptibility. Detailed results of the effects of palmitoylation genes on IA are released in Table S4.

### 3.2 Sensitivity Analysis

Sensitivity analysis is necessary to eliminate the influence of confounding factors on the assessment of causal association. Cochran's *Q* test results showed that all *p* values were greater than 0.05, indicating no heterogeneity. The scatter‐plots of MR Analysis of the six genes were shown in the Figures S1A–S6A, and the results of funnel plots were Supplementary material 2 S1B‐S6B. MR‐PRESSO outlier test analysis showed that there were no outliers. Although Egger's intercept test suggested potential pleiotropic effects for both ZDHHC24 (intercept = 0.0511, *p* = 0.0331) and ZDHHC7 (intercept = 0.0238, *p* = 0.0368), MR‐PRESSO global tests (*p* > 0.05 for both) demonstrated no significant horizontal pleiotropy for either gene (Table S5). None of the individual SNPs employed introduces bias into the MR estimates, as evidenced by leave‐one‐out analyses (Figures S1D–S6D).

### 3.3 SMR Analysis

**FIGURE 2:**
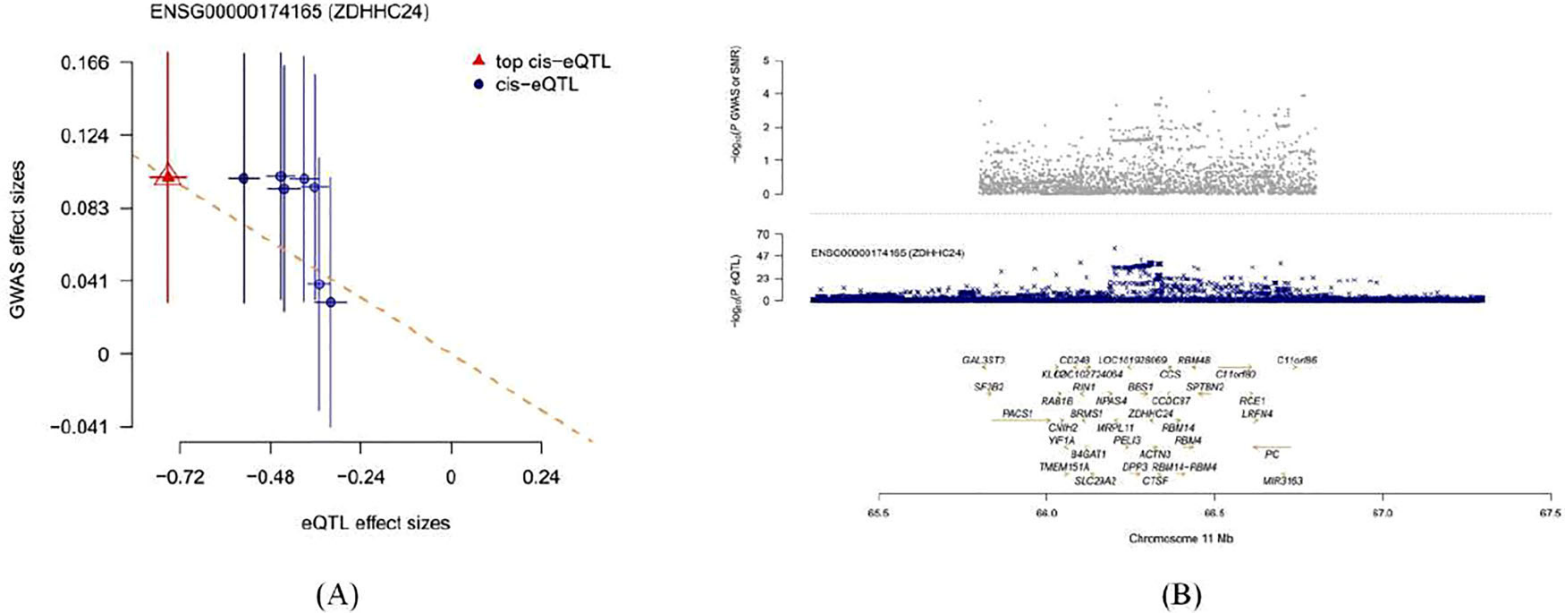
(A) The SNP effect plot of SMR; (B) The SMR locusplot.

The SMR analysis revealed that only the ZDHHC24 gene exhibited a significant association with IA risk (b‐SMR = 0.5391, *p* = 0.0102), suggesting a potential promotive role of ZDHHC24 in the pathogenesis of IA. Importantly, the HEIDI test showed no evidence of pleiotropy‐driven false positive associations (*p* = 0.0648), further supporting a robust causal relationship between ZDHHC24 and IA. The SNP effect plot and chromosomal positional diagram for ZDHHC24 visually represent the top‐associated SNP and its genomic location (Figure 2). Detailed analysis results can be found in Table S6.

### 3.4 DNA Methylation Sites Regulate Palmitoylation Genes Affecting IA

We initiated our analysis by identifying 21 methylation sites associated with the ZDHHC24 gene through the EWAS Data Hub, followed by comprehensive data extraction for all sites from the GoDMC database (Table S7). Following stringent quality control, seven methylation sites (cg01806972, cg04006266, cg10523820, cg18862171, cg24423806, cg24912904, and cg26041493) with 267 associated SNPs were ultimately selected as IVs to evaluate DNA methylation effects on IA susceptibility (Table S8). All IVs satisfied the robustness criterion (*F*‐statistic > 10), enabling a reliable assessment of each SNP's contribution to IA risk (Table S9). The MR Analysis results of seven DNA methylation sites are shown in Table S10. IVW method confirmed that these seven sites had significant causal association with IA susceptibility, among which cg26041493 (OR = 1.1073 95% CI: 1.0727–1.1431 *p* = 3.22E‐10), cg04006266 (OR = 1.1248 95% CI: 1.0887–1.1622 *p* = 1.63E‐12) and

cg24423806 (OR = 1.0577 95% CI: 1.0350–1.0809 *p* = 4.04E‐07) was associated with an increased risk of IA. while

cg10523820 (OR = 0.5585 95% CI: 0.4733–0.6592 *p* = 5.53E‐12),

cg18862171 (OR = 0.7086 95% CI: 0.6257–0.8026 *p* = 5.88E‐08),

cg24912904 (OR = 0.3884 95% CI: 0.2666–0.5659 *p* = 8.45E‐07) and

cg01806972 (OR = 0.7213 95% CI: 0.6572–0.7916 *p* = 5.89E‐12) was associated with a reduced risk of IA.

We then evaluated the effect of seven DNA methylation sites strongly associated with IA susceptibility on the expression of ZDHHC24. IVW assay confirmed six of the seven sites (cg01806972, cg26041493, cg04006266, cg10523820, cg18862171, and cg24423806) were involved in gene expression regulation, and the final cg01806972 (OR = 0.5777 95% CI: 0.5236–0.6374 *p* = 8.22E‐28), cg10523820 (OR = 0.4551 95% CI: 0.4210–0.4920 *p* = 2.57E‐87) and

cg18862171 (OR = 0.5633 95% CI: 0.4724–0.6717, *p* = 1.64E‐10) caused a decrease in gene expression, while

cg26041493 (OR = 1.2755 95% CI: 1.2227–1.3306, *p* = 1.75E‐29)

cg04006266 (OR = 1.1637 95% CI: 1.1273–1.2012, *p* = 7.49E‐21) and

cg24423806 (OR = 1.1393 95% CI: 1.1049 to 1.1747 *p* = 7.00E‐17) caused an increase in gene expression. The DNA methylation site cg24912904 was discarded because there were not enough IVs available. Detailed results are provided in Tables S11–S13.

We systematically calculated: (1) the total effect of DNA methylation on IA susceptibility, (2) the direct effect after excluding ZDHHC24 expression interference, and (3) the mediation effect of DNA methylation influencing IA risk through ZDHHC24 expression regulation. The proportion of mediation effects was subsequently calculated (Table 1). Additional details of the mediation analyses are provided in Table S14.

**TABLE 1:** Comprehensive mediation analysis of DNA methylation regulating ZDHHC24 expression on IA risk.

Exposure	Mediating factors	Outcome	nsnp	Total effect	Beta1	Beta2	Effect of mediation	Direct effect	*p*	proportion of mediating effect
cg01806972	ZDHHC24	Intracranial aneurysms	9	−0.3267	−0.5487	0.1976	−0.1084	−0.2183	0.0013	0.3319
cg10523820	ZDHHC24	Intracranial aneurysms	7	−0.5825	−0.7871	0.1976	−0.1556	−0.4269	< 0.0001	0.2671
cg18862171	ZDHHC24	Intracranial aneurysms	7	−0.3444	−0.5739	0.1976	−0.1134	−0.2310	0.0396	0.3293
cg26041493	ZDHHC24	Intracranial aneurysms	53	0.1020	0.2433	0.1976	0.0481	0.0539	0.0176	0.4716
cg04006266	ZDHHC24	Intracranial aneurysms	93	0.1176	0.1516	0.1976	0.0300	0.0877	0.1287	0.2546
cg24423806	ZDHHC24	Intracranial aneurysms	92	0.0561	0.1304	0.1976	0.0258	0.0303	0.1901	0.4595

### 3.5 Transcriptomics Analyses

**FIGURE 3:**
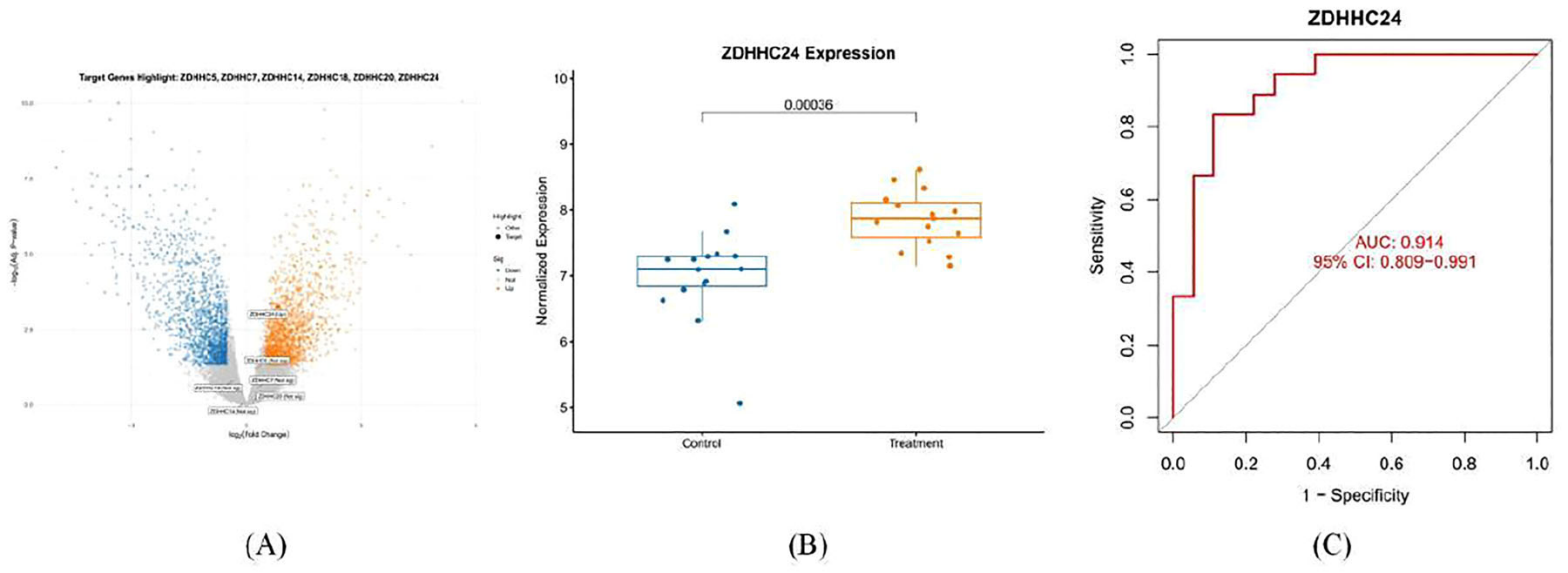
(A) Volcano plots portraying the distribution of differentially expressed genes (DEG) (logFC = 0.585); (B) Box plots depict the differential gene expression profiles of the ZDHHC24 gene in different cohorts in the GSE75436 dataset (*p* = 0.00036); (C) Diagnostic performance of the ZDHHC24 gene (AUC = 0.914).

Through a comprehensive screening of the GEO database, we identified and utilized datasets GSE26969 and GSE75436 for our analysis. In dataset GSE75436, we confirmed significant differential expression of ZDHHC24 between IA patients and normal vascular controls, with marked upregulation of ZDHHC24 in the IA group (*p* < 0.001). Notably, ZDHHC24 demonstrated excellent diagnostic performance in distinguishing IA cases from controls, achieving an area under the curve (AUC) of 0.914 in receiver operating characteristic (ROC) analysis (Figure 3). Gene Ontology (GO) and Kyoto Encyclopedia of Genes and Genomes (KEGG) enrichment analyses revealed significant pathway associations (false discovery rate < 0.05), with complete results and visualization presented in Figure S7A–E.

In the IA‐versus‐normal vascular control comparison, a gene‐based nomogram revealed a robust and stable contribution of ZDHHC24 to IA risk prediction (Figure S9A,B). We therefore stratified the integrated GSE26969 and GSE75436 cohort into high‐ and low‐ZDHHC24 expression subsets; the top five significantly enriched gene functions and pathways within each stratum are presented in Figure S8A–D. To further clarify the discrete role of ZDHHC24 in IA pathogenesis, we reanalyzed the murine intracranial aneurysm scRNA‐seq dataset GSE193533. Palmitoylation‐related genes displayed marked cell‐type specificity, with ZDHHC24 transcripts predominantly localizing to fibroblasts and vascular endothelial cells (Figure S10A–C). This bimodal distribution was independently corroborated in the human embryo forebrain atlas (SRA646572) via the PanglaoDB portal (Figure S11A,B), collectively implicating ZDHHC24 as a gene intimately coupled to endothelial–fibroblast biology.

## 4 Discussion

This study employed two‐sample MR analysis, SMR analysis, mediation analysis, and transcriptome analysis to investigate the relationship between palmitoylation‐related genes and unruptured IA risk. Our results demonstrate that ZDHHC24 expression was significantly higher in IA patients than in normal controls, and this elevated expression showed a significant causal association with increased IA risk mediated by DNA methylation. Sensitivity analyses confirmed the robustness of our findings. This MR study provides new insights into IA pathophysiology, and further research may help guide early diagnosis and precision treatment of IA.

Our two‐sample MR and SMR analyses confirmed a significant causal relationship between elevated ZDHHC24 expression and increased risk of IA. Subsequent transcriptomic profiling demonstrated markedly higher ZDHHC24 expression levels in IA patients compared with normal controls (*p* < 0.001). ZDHHC24 (Zinc Finger DHHC‐Type Containing 24 or Zinc Finger DHHC‐Type Palmitoyltransferase 24) is a protein‐coding gene. Predicted to enable protein–cysteine S‐palmitoyltransferase activity (Zhang et al. 2023). Previous studies have rarely implicated ZDHHC24 as a pathogenic gene, though seminal work by Jianping Guo et al. revealed through RNA‐sequencing analysis of ZDHHC24^−/−^ murine liver tissues that ZDHHC24 deficiency markedly impaired hepatic metabolic function, ZDHHC24‐mediated palmitoylation and activation of AKT is important for their function in promoting nonalcoholic fatty liver disease (NAFLD) and hepatocellular carcinoma (Bu, Zhang, et al. 2024). Notably, their research further identified ZDHHC24 as the most efficient enzyme promoting mitochondrial antiviral signaling protein (MAVS) palmitoylation and activation. Specifically, ZDHHC24 catalyzes palmitic acid‐induced MAVS palmitoylation, oligomerization, and subsequent activation, thereby potently enhancing innate immune responses (Bu, Wang, et al. 2024). However, the precise role of ZDHHC24 in the pathogenesis of IA remains inadequately understood.

Our DNA methylation and mediation analyses identified four key methylation sites (cg01806972, cg10523820, cg18862171, and cg26041493) that regulate ZDHHC24 gene expression and contribute to IA risk modulation. Among these, cg01806972, cg10523820, and cg18862171 were significantly associated with downregulation of ZDHHC24 expression, while cg26041493 was associated with upregulation of ZDHHC24 expression. To our knowledge, the functional roles and mechanistic contributions of these four methylation sites in IA pathogenesis have not been previously reported in methylation studies. DNA methylation predominantly occurs at CpG islands within gene promoter regions and is typically associated with transcriptional silencing (Jones 2012). The study by Lin et al. (2025) reported that methylation sites cg06382664, cg04295144, cg18850434, cg08770935, and cg21810604 modulate the risk of unruptured IA and aSAH through regulating the expression of RELT and ICAM5 genes. Elisa Fernández‐Pérez et al. (2024) and Macias‐Gómez et al. (2024) further demonstrated that DNA methylation not only participates in aSAH‐induced vasospasm, but also exhibits an independent association between DNA methylation‐based biological age and 12‐month post‐hemorrhage mortality. Furthermore, Maimaiti et al. (2023) elucidated the potential role of DNA methylation in IA pathogenesis through integrative multi‐omics analyses. Although familial aggregation confers a marked heritable predisposition—manifest as a stepwise elevation in IA and aSAH risk with the number of affected first‐degree relatives—published heritability analyses indicate that common genetic variants explain only ∼40% of the total variance, leaving a substantial residual liability unaccounted for (Rinkel and Ruigrok 2022; Bakker and Ruigrok 2021). Environmental determinants such as cigarette smoking is therefore posited to precipitate the observed familial excess, plausibly via epigenetic reprogramming (Chou 2021). Work by Wang, Zhou, et al. (2021) demonstrated that chronic tobacco‐smoke exposure induces DNA hypermethylation within the PTBP1 promoter, suppressing PTBP1 transcription and thereby contributing to intracranial aneurysm pathogenesis. In addition, the principal risk factors for IA formation and rupture—female sex and arterial hypertension—have themselves been shown to remodel the epigenome and thereby modulate IA susceptibility (Fernández‐Pérez et al. 2024; Soriano‐Tárraga et al. 2021; Soriano‐Tárraga et al. 2018). These findings collectively suggest that DNA methylation may substantially contribute to IA pathobiology via regulation of disease‐associated gene expression through multiple mechanistic pathways. Our study represents the first investigation of the palmitoylation gene ZDHHC24 and its associated methylation sites in IA risk. The development of inhibitors targeting these palmitoyltransferases may offer novel strategies for IA prevention and early intervention, although significant translational challenges remain. Alternatively, modulation of upstream regulators of these palmitoyltransferases could also yield therapeutic potential and warrants further exploration.

Our study provides robust causal evidence for the role of palmitoylation genes in IA pathogenesis through an integrated analytical framework with multiple methodological strengths. First, we employed MR to overcome the high costs and practical limitations of randomized controlled trials while effectively minimizing confounding through stringent genetic instrument selection (*F*‐statistic > 10). Second, we implemented a multi‐method approach combining two‐sample MR, SMR, and mediation analyses, with all findings further validated through comprehensive sensitivity analyses (MR‐Egger, weighted median) and transcriptomic profiling in IA patient cohorts. Finally, our analyses leveraged the authoritative FinnGen R12 dataset (*n* > 450,000), ensuring both statistical power and clinical validity through its large sample size and rigorous phenotyping. This study represents the first comprehensive investigation of palmitoylation–epigenetic crosstalk in IA, establishing ZDHHC24 as a novel susceptibility gene while simultaneously identifying druggable targets for potential therapeutic intervention. All reported associations survived multiple testing correction (Bonferroni *p *< 0.05) and met stringent sensitivity criteria, including Steiger filtering and LD‐pruning (*r* ^2^ < 0.001), underscoring the reliability of our conclusions.

While our study provides novel insights into the role of palmitoylation genes in IA pathogenesis, several limitations should be acknowledged. First, our analyses are grounded in large‐scale population cohorts and therefore capture only a cross‐sectional, time‐aggregated snapshot of the population under the prevailing conditions; they cannot be used to infer how the influence of any given exposure on disease risk evolves over time. Second, although MR methods reduce confounding, residual environmental factors (e.g., temperature, humidity) may still influence the results. Third, although the use of individuals of European ancestry exclusively (from the FinnGen R12 dataset) was necessary to mitigate population stratification, this constraint inherently limits the generalizability of our findings to non‐European populations. Allele frequencies, linkage‐disequilibrium architectures, environmental exposures (e.g., tobacco smoke, ambient air pollution, heavy‐metal contaminants), and lifestyle factors (e.g., circadian disruption, high‐fat diets) differ substantially across ancestries, any of which may modulate both the effect sizes and clinical utility of the identified variants. Consequently, prospective translation into broad clinical practice mandates rigorous replication and validation in large, ancestrally diverse cohorts. Another major limitation is our reliance on blood‐derived QTL data. Although we attempted to evaluate the cross‐tissue relevance of our ZDHHC24 lead variants using the GTEx database, we were only able to replicate our findings in aortic tissue (detailed results of the validation analysis for ZDHHC24 gene expression in aortic tissue and IA risk are presented in Table S15); direct validation in human intracranial arteries or aneurysm tissue remains lacking. This underscores the potential for tissue‐specific effects and cautions against overinterpretation. Consequently, our results should be viewed as generating a robust hypothesis that awaits direct validation in disease‐relevant cerebrovascular tissues in future studies. Most importantly, while our integrated omics approach identifies promising mechanistic links, experimental validation through animal models and randomized controlled trials will be essential to confirm these findings and assess their clinical applicability. These limitations highlight important considerations for interpreting our results and directions for future research.

Epigenetics is a flourishing field with still little exploration in vascular neurosurgery. In light of our findings and the methodological limitations identified, we propose the following future directions to advance epigenetic research beyond the constraints of previous studies. First, large‐scale EWAS with sufficient statistical power are essential to enable rigorous adjustments and yield robust findings; such efforts, bolstered by technological advances, will uncover novel methylation biomarkers that can serve as therapeutic targets or tools for early detection and prognosis of IA and aSAH. Second, integrating EWAS data with multi‐omics layers—genomics, transcriptomics, proteomics—will deepen our understanding of the molecular pathways disrupted in these diseases and facilitate the development of targeted therapies. Third, systematic investigation of mQTLs will elucidate how genetic susceptibility interacts with environmental exposures such as smoking and hypertension, thereby refining risk prediction and identifying modifiable gene–environment interfaces. Finally, as underscored by the limitations of the current work, rigorous validation in cellular and animal models is indispensable, and future studies should prioritize longitudinal cohorts that track epigenetic changes from pre‐disease through progression; this longitudinal–experimental pipeline will constitute the central focus of our subsequent research and will underpin personalized therapeutic strategies aligned with individual epigenomic profiles, ultimately advancing precision medicine for IA and aSAH.

## 5 Conclusion

Our study demonstrates through multi‐omics analyses that DNA methylation modulates IA risk by regulating ZDHHC24 expression. These findings elucidate potential mechanisms underlying IA pathogenesis and underscore the pivotal role of epigenetics in cerebrovascular disorders. Further in‐depth investigations will contribute to enhanced comprehension and clinical management of IA.

## Author Contributions

H.Y. is responsible for conceptualization, data curation, formal analysis, investigation, methodology, and project administration; also contributed to validation, and drafted the original manuscript. C.L. engaged in formal analysis, investigation, and contributed to drafting the original manuscript. X.F., J.L., C.H., and M.H. were involved in conceptualization, data curation, investigation, and provided critical review and editing of the manuscript. C.D. played a role in conceptualization, data curation, formal analysis, secured funding, conducted investigation, developed methodology, and managed project administration; also responsible for validation, and critically reviewed and edited the manuscript. All authors have reviewed and provided feedback on the manuscript and have approved the final version.

## Funding

This research was supported by Special Funds for the Cultivation of Guangdong College Students’ Scientific and Technological Innovation (pdjh2025bk052).

## Ethics Statement

All data analyzed in this study were obtained from publicly available databases. Ethical approval was obtained for each cohort, and informed consent was obtained from all participants prior to participation. No additional ethical approval was required for this study.

## Conflicts of Interest

The authors declare no conflicts of interest.

## Supporting information



Supplementary Table S1 palmitoylation candidate genes
Supplementary Table S2 IVs for Palmitoylation genes
Supplementary Table S3 MR Effects for each IV
Supplementary Table S4 Effect of palmitoylation gene on IA
Supplementary Table S5 The sensitivity analysis for IA susceptibility
Supplementary Table S6 The result of SMR
Supplementary Table S7 Information of 21 methylation sites associated with ZDHHC24
Supplementary Table S8 IVs for DNA methylation sites
Supplementary Table S9 MR Effects for each IV
Supplemental Table S10 Effects of DNA methylation on susceptibility to IA
Supplementary Table S11 IVs used to assess how methylation sites affect gene expression
Supplementary Table S12 MR Effects for each IV
Supplemental Table S13 Effects of DNA methylation on expression of ZDHHC24
Supplementary Table 14 Results of mediation analysis
Supplementary Table S15 Causal association between ZDHHC24 and IA in aortic tissue

Supplementary Fig. S1 Scatter plot (A), funnel plot (B), forest plot (C) and leave‐one‐out sensitivity analysis (D) of SNPs associated with ZDHHC5 on IA
Supplementary Fig. S2 Scatter plot (A), funnel plot (B), forest plot (C) and leave‐one‐out sensitivity analysis (D) of SNPs associated with ZDHHC7 on IA
Supplementary Fig. S3 Scatter plot (A), funnel plot (B), forest plot (C) and leave‐one‐out sensitivity analysis (D) of SNPs associated with ZDHHC14 on IA
Supplementary Fig. S4 Scatter plot (A), funnel plot (B), forest plot (C) and leave‐one‐out sensitivity analysis (D) of SNPs associated with ZDHHC18 on IA
Supplementary Fig. S5 Scatter plot (A), funnel plot (B), forest plot (C) and leave‐one‐out sensitivity analysis (D) of SNPs associated with ZDHHC20 on IA
Supplementary Fig. S6 Scatter plot (A), funnel plot (B), forest plot (C) and leave‐one‐out sensitivity analysis (D) of SNPs associated with ZDHHC24 on IA
Supplementary Fig. S7 Bar plot of GO enrichment analysis (A), bubble plot of GO enrichment analysis (B), circle plot of GO enrichment analysis (C), bar plot of KEGG enrichment analysis (D), bubble plot of KEGG enrichment analysis (E), ROC curve of the ZDHHC24 gene in data set GSE26969 to distinguish IA disease group and control group with AUC = 1 (F)
Supplementary Fig. S8 Gene functions analyzed by GSEA enrichment in the control group (A), gene functions analyzed by GSEA enrichment in the IA group (B), gene pathways analyzed by GSEA enrichment in the control group (C), gene pathways analyzed by GSEA enrichment in the IA group (D)
Supplementary Fig. S9 The effect of palmitoylation genes on disease risk (A); Predicted probability (B)
Supplementary Fig. S10 Schematic representation of cell grouping (A); Schematic illustration of cell type annotation (B); Schematic representation of the distribution of the featured genes in the cell population (C); Mouse IA model dataset GSE193533
Supplementary Fig. S11 Schematic representation of cell grouping (A); Schematic illustration of cell type annotation (B); Human embryo forebrain dataset SRA646572

## Data Availability

The complete research datasets generated in this study have been permanently archived and publicly released via the Zenodo repository under the persistent identifier: https://doi.org/10.5281/zenodo.15767468. This DOI‐resolvable link provides access to all supplementary materials, including raw data, processed analytical files, and associated metadata.
