# Supplementary material for: DNA Methylation‐Regulated ZDHHC24 Exacerbates the Risk of Intracranial Aneurysms
Source: Brain Behav. 2026 Apr 6;16(4):e71213. doi: 10.1002/brb3.71213 (PMC13053309; doi:10.1002/brb3.71213)
Supplement: Supplementary file 2 — Supplementary Fig. S1 Scatter plot (A), funnel plot (B), forest plot (C) and leave‐one‐out sensitivity analysis (D) of SNPs associated with ZDHHC5 on IA Supplementary Fig. S2 Scatter plot (A), funnel plot (B), forest plot (C) and leave‐one‐out sensitivity analysis (D) of SNPs associated with ZDHHC7 on IA Supplementary Fig. S3 Scatter plot (A), funnel plot (B), forest plot (C) and leave‐one‐out sensitivity analysis (D) of SNPs associated with ZDHHC14 on IA Supplementary Fig. S4 Scatter plot (A), funnel plot (B), forest plot (C) and leave‐one‐out sensitivity analysis (D) of SNPs associated with ZDHHC18 on IA Supplementary Fig. S5 Scatter plot (A), funnel plot (B), forest plot (C) and leave‐one‐out sensitivity analysis (D) of SNPs associated with ZDHHC20 on IA Supplementary Fig. S6 Scatter plot (A), funnel plot (B), forest plot (C) and leave‐one‐out sensitivity analysis (D) of SNPs associated with ZDHHC24 on IA Supplementary Fig. S7 Bar plot of GO enrichment analysis (A), bubble plot of GO enrichment analysis (B), circle plot of GO enrichment analysis (C), bar plot of KEGG enrichment analysis (D), bubble plot of KEGG enrichment analysis (E), ROC curve of the ZDHHC24 gene in data set GSE26969 to distinguish IA disease group and control group with AUC = 1 (F) Supplementary Fig. S8 Gene functions analyzed by GSEA enrichment in the control group (A), gene functions analyzed by GSEA enrichment in the IA group (B), gene pathways analyzed by GSEA enrichment in the control group (C), gene pathways analyzed by GSEA enrichment in the IA group (D) Supplementary Fig. S9 The effect of palmitoylation genes on disease risk (A); Predicted probability (B) Supplementary Fig. S10 Schematic representation of cell grouping (A); Schematic illustration of cell type annotation (B); Schematic representation of the distribution of the featured genes in the cell population (C); Mouse IA model dataset GSE193533 Supplementary Fig. S11 Schematic representation of cell grouping (A); Schematic illust [file BRB3-16-e71213-s002.docx]

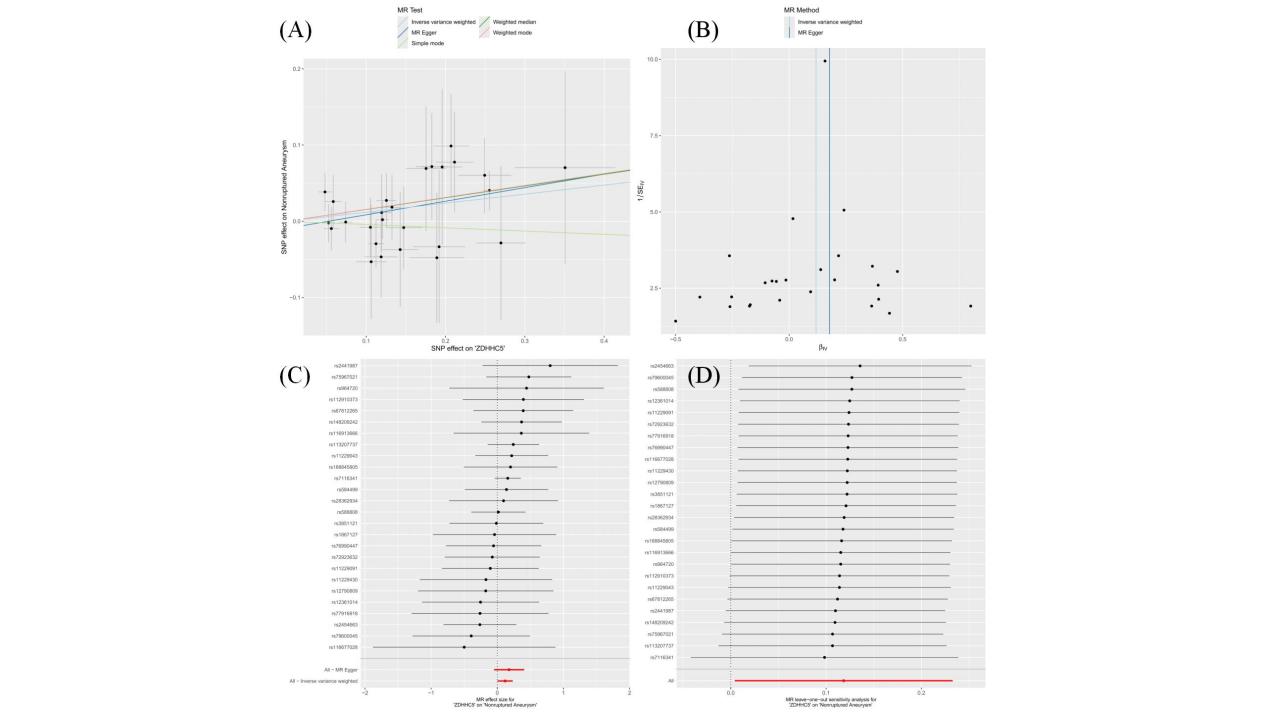


Supplementary Fig. S1 Scatter plot (A), funnel plot (B), forest plot (C) and leave-one-out sensitivity analysis (D) of SNPs associated with ZDHHC5 on IA


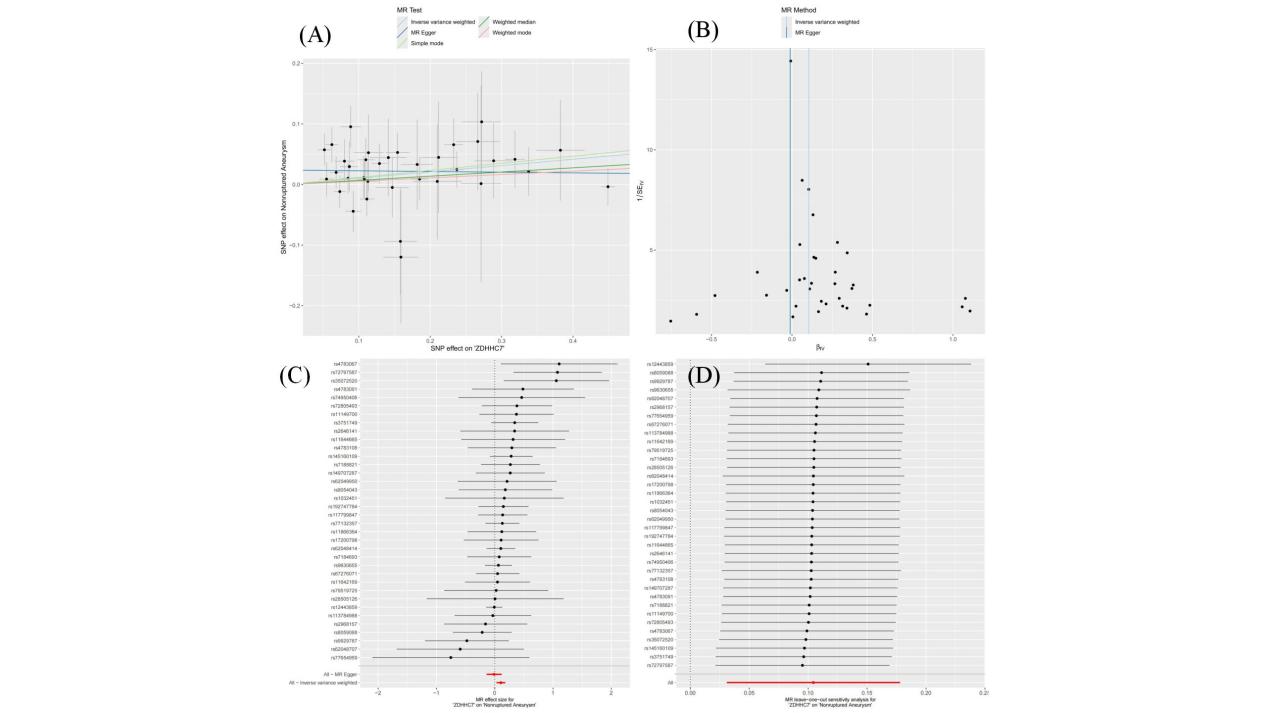


Supplementary Fig. S2 Scatter plot (A), funnel plot (B), forest plot (C) and leave-one-out sensitivity analysis (D) of SNPs associated with ZDHHC7 on IA


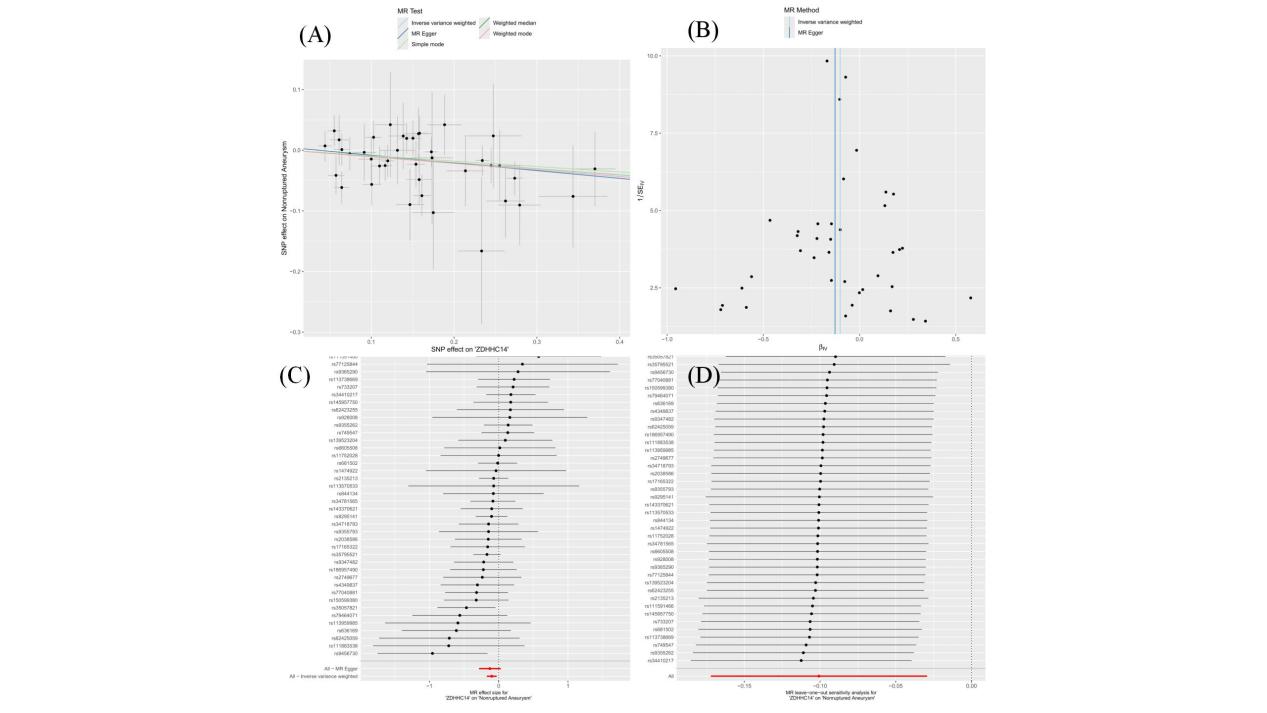


Supplementary Fig. S3 Scatter plot (A), funnel plot (B), forest plot (C) and leave-one-out sensitivity analysis (D) of SNPs associated with ZDHHC14 on IA


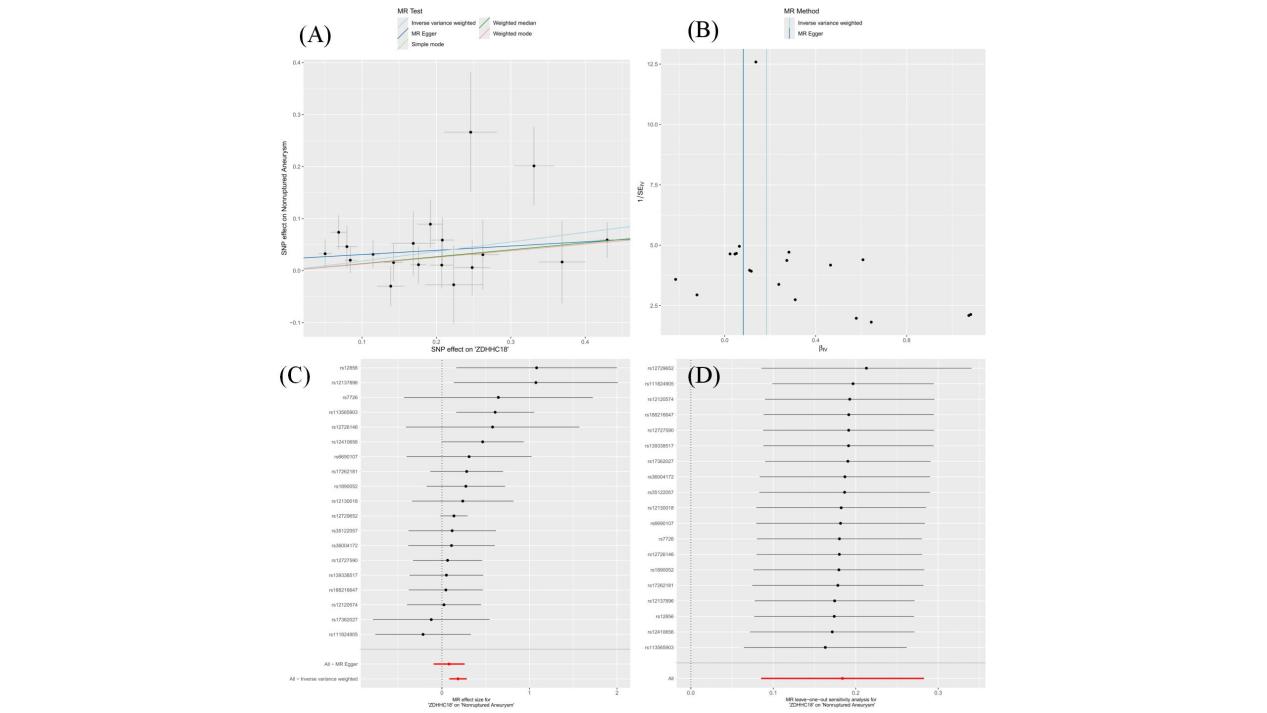


Supplementary Fig. S4 Scatter plot (A), funnel plot (B), forest plot (C) and leave-one-out sensitivity analysis (D) of SNPs associated with ZDHHC18 on IA


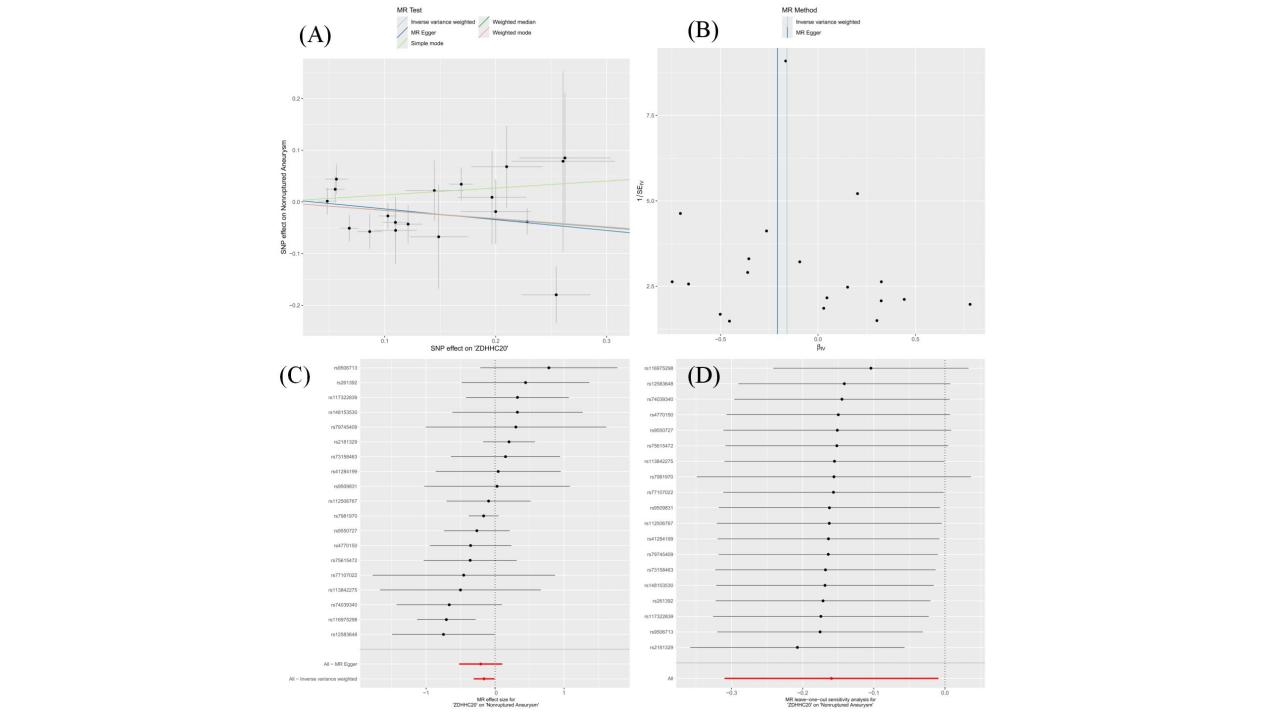


Supplementary Fig. S5 Scatter plot (A), funnel plot (B), forest plot (C) and leave-one-out sensitivity analysis (D) of SNPs associated with ZDHHC20 on IA


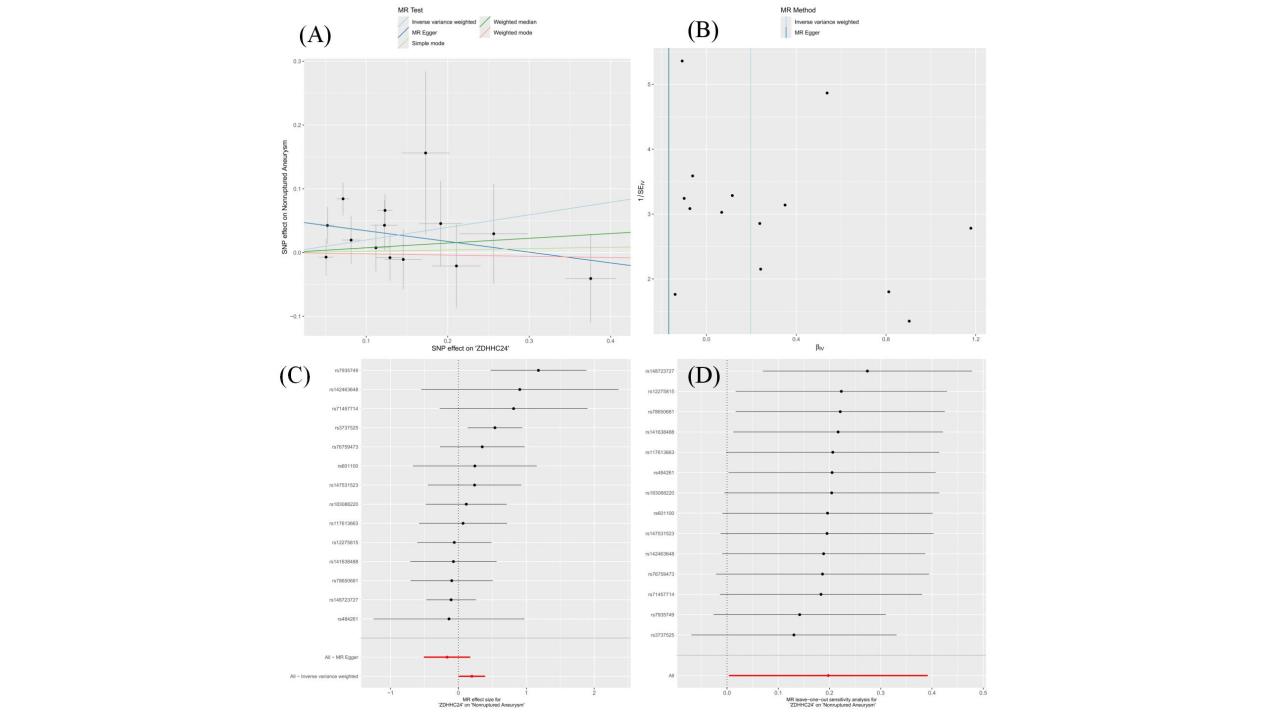


Supplementary Fig. S6 Scatter plot (A), funnel plot (B), forest plot (C) and leave-one-out sensitivity analysis (D) of SNPs associated with ZDHHC24 on IA


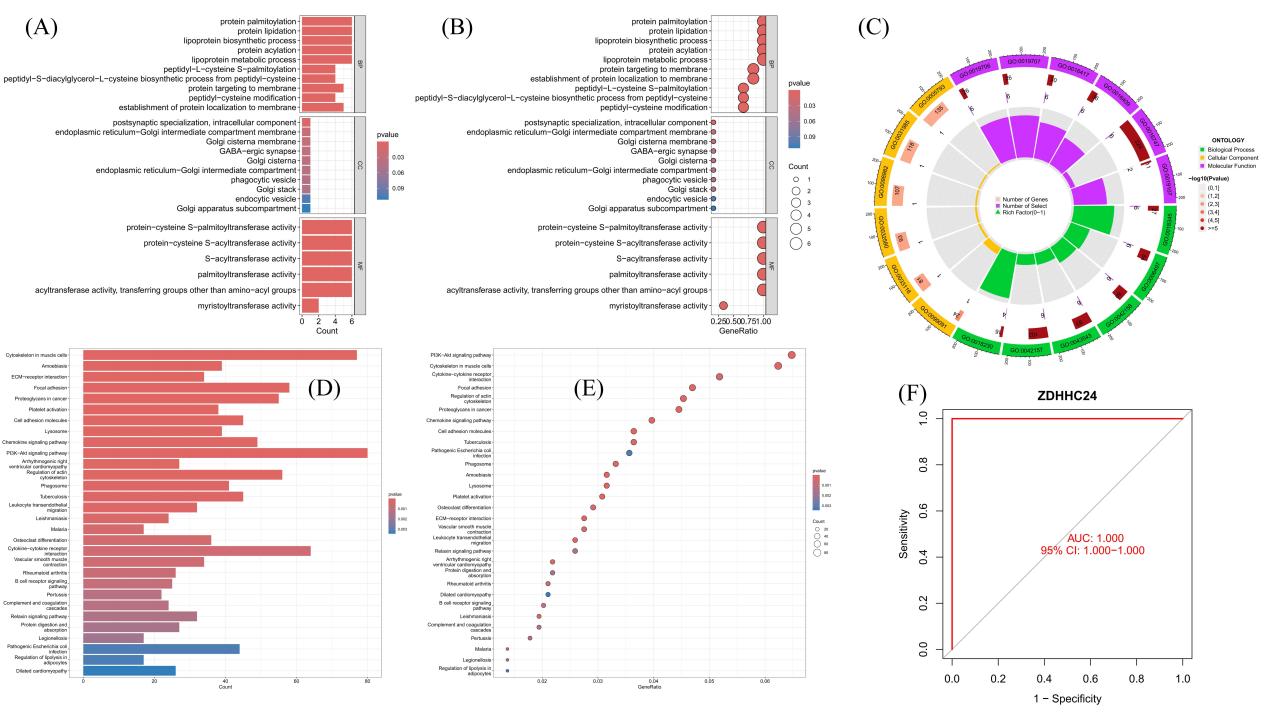


Supplementary Fig. S7 Bar plot of GO enrichment analysis (A), bubble plot of GO enrichment analysis (B), circle plot of GO enrichment analysis (C), bar plot of KEGG enrichment analysis (D), bubble plot of KEGG enrichment analysis (E), ROC curve of the ZDHHC24 gene in data set GSE26969 to distinguish IA disease group and control group with AUC = 1 (F)


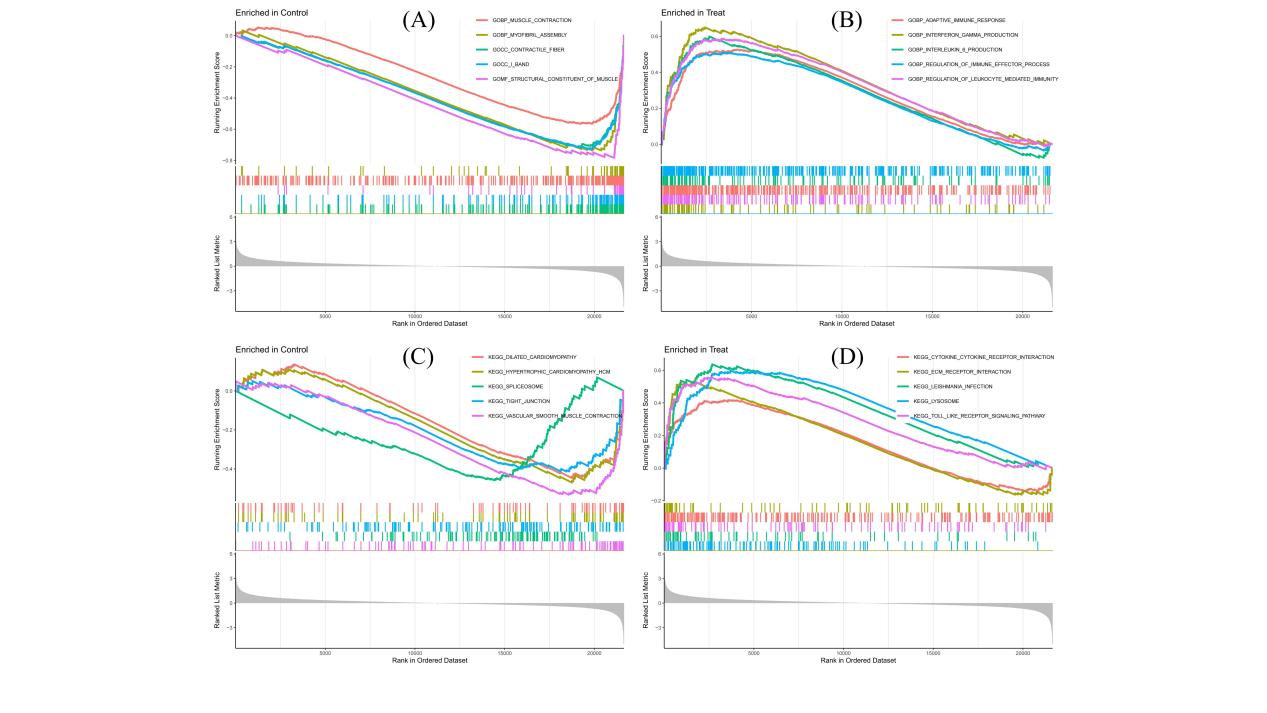


Supplementary Fig. S8 Gene functions analyzed by GSEA enrichment in the control group (A), gene functions analyzed by GSEA enrichment in the IA group (B), gene pathways analyzed by GSEA enrichment in the control group (C), gene pathways analyzed by GSEA enrichment in the IA group (D)


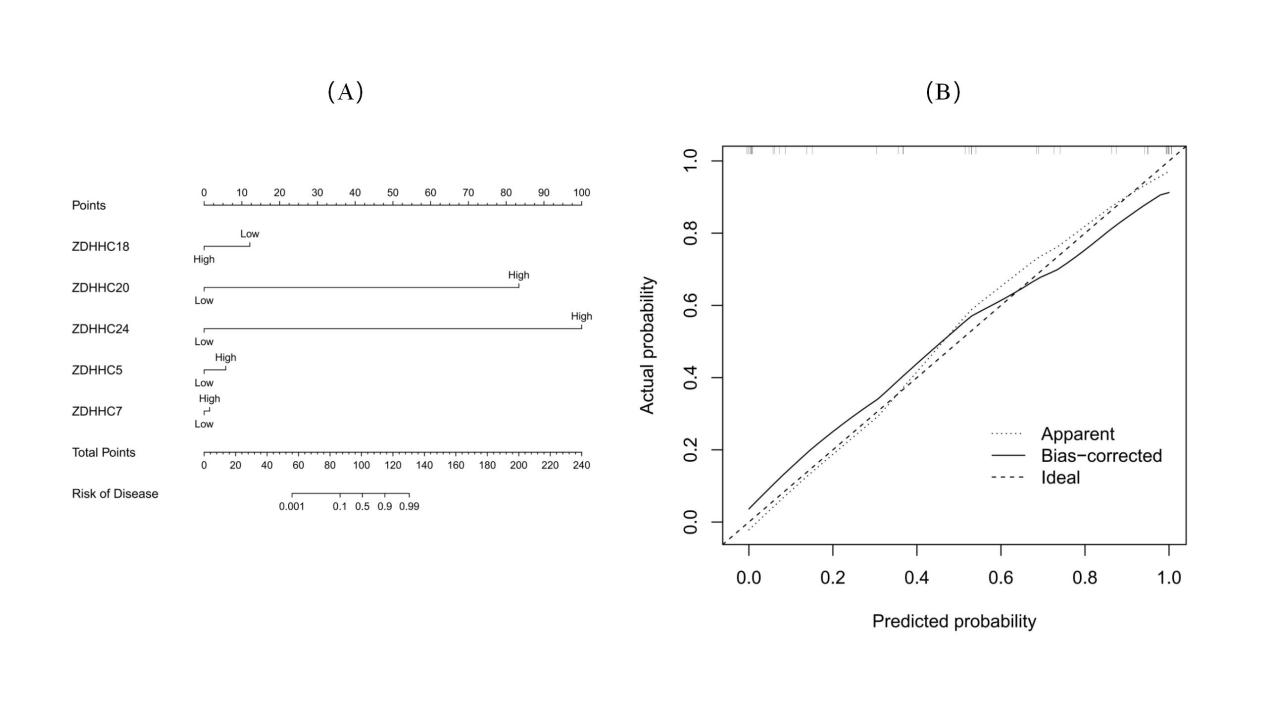


Supplementary Fig. S9 The effect of palmitoylation genes on disease risk (A); Predicted probability (B)


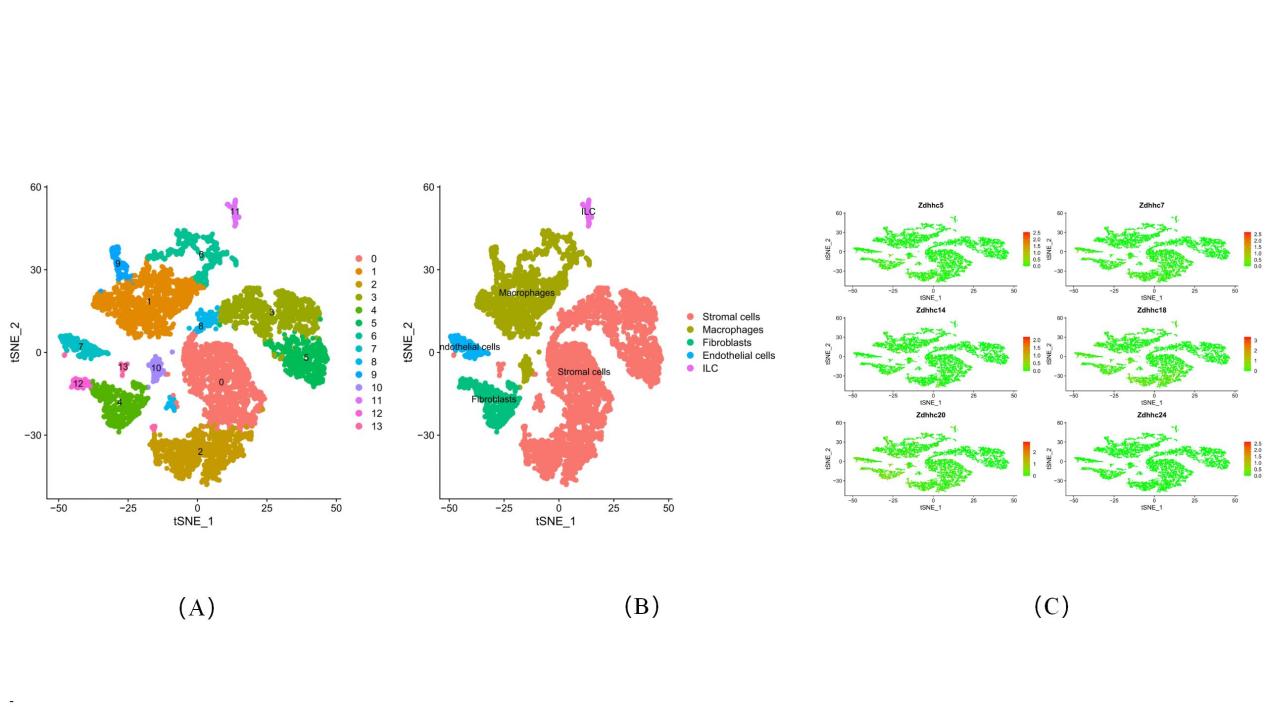


Supplementary Fig. S10 Schematic representation of cell grouping (A); Schematic illustration of cell type annotation (B); Schematic representation of the distribution of the featured genes in the cell population (C); Mouse IA model dataset GSE193533


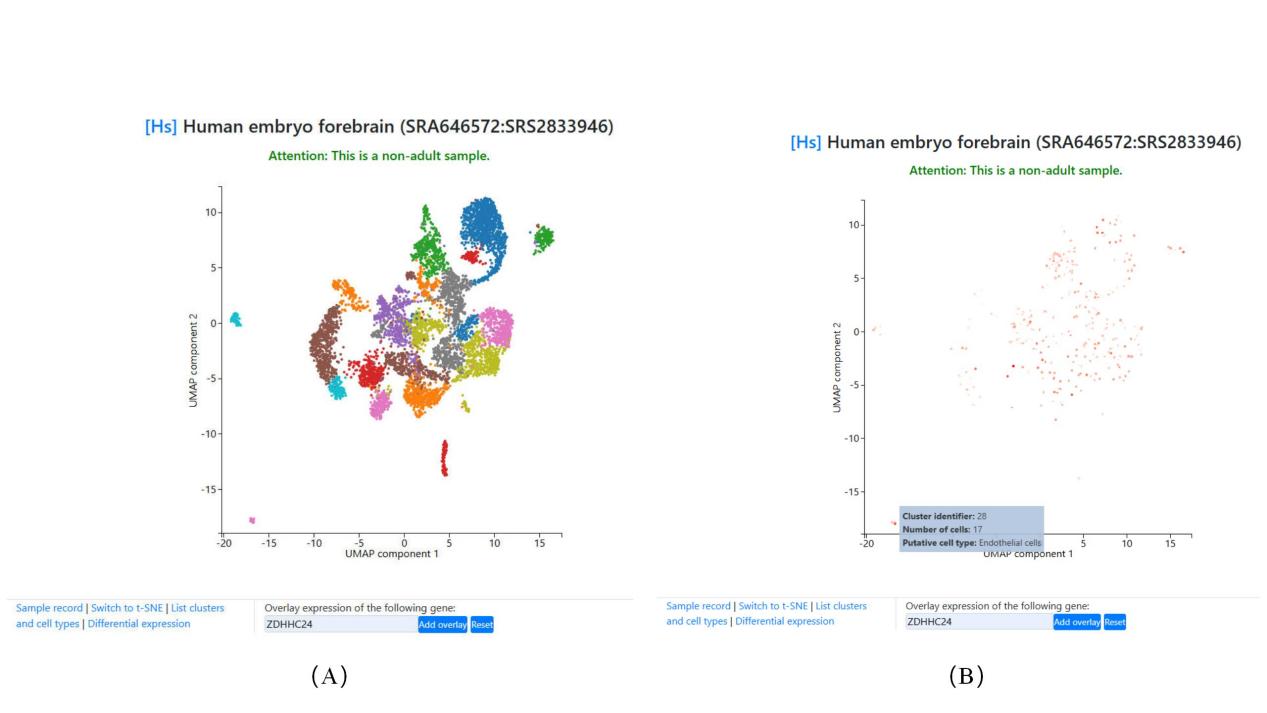


Supplementary Fig. S11 Schematic representation of cell grouping (A); Schematic illustration of cell type annotation (B); Human embryo forebrain dataset SRA646572
